# Seminal Vesicle Mass Fistulising to the Rectum: A Rare Urological Presentation of Lung Cancer Metastasis

**DOI:** 10.3390/reports9030213

**Published:** 2026-07-04

**Authors:** Margarida André, Francisco Vara-Luiz, Luísa Moreira, João Paulo Rosa, Miguel Carvalho

**Affiliations:** 1 Urology Department, Hospital Garcia de Orta, 2805-267 Almada, Portugal; 2 Gastroenterology Department, Hospital Garcia de Orta, 2805-267 Almada, Portugal; 3 Aging Lab, Egas Moniz Center for Interdisciplinary Research (CiiEM), Egas Moniz School of Health and Science, 2829-511 Almada, Portugal

**Keywords:** lung cancer metastasis, seminal vesicle, rectal fistula, urological oncology, rare metastasis

## Abstract

Metastatic involvement of the male genitourinary tract by lung cancer is exceedingly rare. We report a 56-year-old man with metastatic lung adenocarcinoma (initial stage T3N2M1b) under pembrolizumab, who presented with severe pelvic pain. Pelvic magnetic resonance imaging and computed tomography demonstrated a large mass with an imaging epicentre favouring the left seminal vesicle, involving the prostate and fistulising to the distal rectum, without pelvic ascites or peritoneal disease. A total PSA of 0.81 ng/mL and a previous negative prostate biopsy made a primary prostatic malignancy less likely. Biopsy of the rectal component revealed a poorly differentiated carcinoma with an immunophenotype (CK7+, TTF-1+, p40−, CDX2−, NKX3.1−, PAX8−) consistent with metastatic adenocarcinoma of pulmonary origin. The patient underwent palliative pelvic radiotherapy, with improvement of pelvic pain; he subsequently developed pneumaturia and faecaluria and died eight months later from disease progression. Seminal vesicle metastasis from lung carcinoma has been reported previously; to our knowledge, however, this is the first report presenting with rectal fistulisation. This case highlights a diagnostically challenging presentation and the need to consider metastatic disease when evaluating atypical seminal vesicle masses in oncological patients.

Lung cancer is one of the most common malignancies worldwide and a leading cause of cancer-related mortality, with most patients diagnosed at an advanced stage [1]. Distant metastases most frequently involve the liver, adrenal glands, bone and brain, whereas metastatic involvement of the male genitourinary tract is extremely rare [2]. Primary tumours of the seminal vesicles are uncommon, and secondary involvement usually results from direct invasion by adjacent pelvic organs; metastasis to the seminal vesicles is exceptionally rare, with isolated reports from hepatocellular, colorectal and lung primaries [3,4,5].

A 56-year-old man with metastatic pulmonary adenocarcinoma, under pembrolizumab, staged at diagnosis as T3N2M1b—a 5.5 cm necrotic primary in the posterior right lower lobe with a smaller apical nodule, a 3.9 cm left upper-lobe lesion, high right retrotracheal nodal disease, and a 3 cm right adrenal metastasis—presented with severe pelvic pain. Pelvic CT showed a lobulated midline mass in the topography of the seminal vesicles, interposed between the bladder floor and the rectum, containing thick fluid and gas, with a defect in the anterior rectal wall and communication with the bladder floor (Figure 1A). Pelvic MRI demonstrated a solid pelvic mass whose imaging epicentre favoured the left seminal vesicle, involving the upper prostate and fistulising to the anterior wall of the distal rectum; the prostatic peripheral zone was preserved Figure 1(B1,B2). There was no pelvic ascites and no peritoneal or other intra-abdominal disease. A total PSA of 0.81 ng/mL and a prior prostate biopsy negative for malignancy made a primary prostatic tumour less likely.

Flexible sigmoidoscopy identified a bulky, ulcerated mass in the distal rectum (Figure 1C). Biopsy revealed a poorly differentiated, ulcerated carcinoma with an immunophenotype consistent with metastatic adenocarcinoma of pulmonary origin: CK7+, TTF-1+, p40−, CDX2−, NKX3.1−, PAX8−, GATA3−, SOX10−, CD45−, CD56−, with PD-L1 expression of 90% (Figure 1D–F). This profile supports a pulmonary origin and argues against colorectal, prostatic, urothelial, breast, melanocytic, lymphoid and neuroendocrine primaries.

Taken together, imaging favoured a seminal-vesicle epicentre, with involvement of the adjacent prostate and rectum. Because tissue was obtained only from the rectal component, the seminal vesicle cannot be established as the initial metastatic site and the direction of spread cannot be determined. Possible routes include haematogenous dissemination—plausible given the patient’s widespread metastatic disease, though unproven—as well as lymphatic spread and contiguous extension from an adjacent rectal, prostatic or periprostatic metastatic deposit. The absence of ascites and of other intra-abdominal disease makes peritoneal dissemination less likely.

Following the diagnosis, the patient underwent palliative external pelvic radiotherapy, with improvement of the pelvic pain. Although pneumaturia and faecaluria had not been documented at presentation, both developed after radiotherapy, consistent with progression of the recto-urinary fistula. He died eight months after radiotherapy from progressive disease.

Seminal vesicle metastasis from lung carcinoma has been reported previously [5]; to our knowledge, however, this is the first to present with rectal fistulisation. This case underscores the importance of considering metastatic disease when an atypical seminal vesicle mass is found in a patient with a known malignancy.

## Figures and Tables

**Figure 1:**
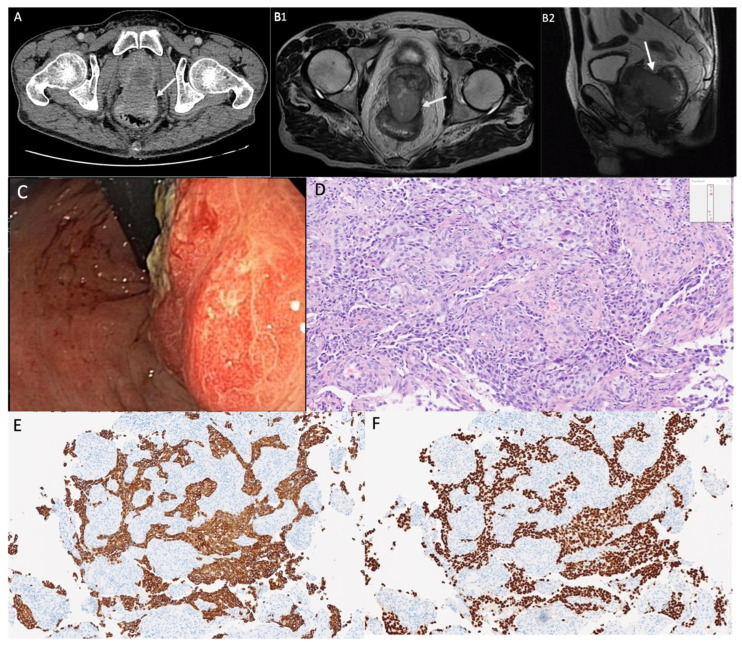
(**A**) Pelvic computed tomography (axial): A lobulated midline mass (arrow, mass) in the topography of the seminal vesicles, containing thick fluid and gas. (**B1**) Pelvic MRI (axial T2-weighted): Solid lesion with imaging epicentre favouring the left seminal vesicle (arrow, mass); the prostatic peripheral zone is preserved. (**B2**) Pelvic MRI (sagittal T2-weighted): The fistulous tract (arrow) and the mass, demonstrating the relationship among the seminal vesicle, prostate and rectum. (**C**) Flexible sigmoidoscopy: Bulky, ulcerated mass in the distal rectum. (**D**) Haematoxylin and eosin: Poorly differentiated carcinoma with solid and acinar growth patterns (original magnification 100×). (**E**,**F**) Immunohistochemistry: Strong positivity for CK7 (**E**) and TTF-1 (**F**), consistent with pulmonary origin (original magnification 40×).

## Data Availability

The original contributions presented in this study are included in the article. Further inquiries can be directed to the corresponding author.
